# Magnetic Lateral Flow Immunoassays

**DOI:** 10.3390/diagnostics10050288

**Published:** 2020-05-08

**Authors:** Amanda Moyano, Esther Serrano-Pertierra, María Salvador, José Carlos Martínez-García, Montserrat Rivas, M. Carmen Blanco-López

**Affiliations:** 1 Department of Physical and Analytical Chemistry & Institute of Biotechnology of Asturias, University of Oviedo, c/ Julián Clavería 8, 33006 Oviedo, Spain; moyanoamanda@uniovi.es (A.M.); serranoesther@uniovi.es (E.S.-P.); 2 Department of Physics & IUTA, University of Oviedo, Campus de Viesques, 33204 Gijón, Spain; salvadormaria@uniovi.es (M.S.); jcmg@uniovi.es (J.C.M.-G.); rivas@uniovi.es (M.R.)

**Keywords:** magnetic nanoparticles, lateral flow immunoassay, magnetic biosensors, optical transduction, magnetic transduction, nanocomposites

## Abstract

A new generation of magnetic lateral flow immunoassays is emerging as powerful tool for diagnostics. They rely on the use of magnetic nanoparticles (MNP) as detecting label, replacing conventional gold or latex beads. MNPs can be sensed and quantified by means of external devices, allowing the development of immunochromatographic tests with a quantitative capability. Moreover, they have an added advantage because they can be used for immunomagnetic separation (IMS), with improvements in selectivity and sensitivity. In this paper, we have reviewed the current knowledge on magnetic-lateral flow immunoassay (LFIA), coupled with both research and commercially available instruments. The work in the literature has been classified in two categories: optical and magnetic sensing. We have analysed the type of magnetic nanoparticles used in each case, their size, coating, crystal structure and the functional groups for their conjugation with biomolecules. We have also taken into account the analytical characteristics and the type of transduction. Magnetic LFIA have been used for the determination of biomarkers, pathogens, toxins, allergens and drugs. Nanocomposites have been developed as alternative to MNP with the purpose of sensitivity enhancement. Moreover, IMS in combination with other detection principles could also improve sensitivity and limit of detection. The critical analysis in this review could have an impact for the future development of magnetic LFIA in fields requiring both rapid separation and quantification.

## 1. Introduction

Point-of-care testing (POCT) devices are diagnostic tools for rapid in-field analysis. They should be simple and accessible to unskilled operators at decentralized settings. They can be classified into several categories such as cell phone-based technologies, paper-based diagnostics tools and lab-on-a-chip-based platforms [1]. The blood glucose meter and the pregnancy test are the most popular examples of this technology [2]. In the last decade, research works have focused on improving sensitivity, quantification and multiplexing [3]. Thus, POCT procedures may be applied in a variety of fields such as clinical diagnosis [4], safety [5], agriculture [6], veterinary [7], drugs [8] and contaminants [9].

Lateral flow immunoassays (LFIA) are widely used as POCT due to their rapidity, simplicity, and low cost. This principle combines a chromatographic system with immunochemical reactions for specific and sensitive detection. Traditionally, LFIAs have been used only as qualitative screening tests, with a visual signal for positive/negative test. The outbreak of the coronavirus disease (COVID-19) is the most recent example of the relevance of these devices in rapid screening and monitoring [10].

The test consists of four components: sample pad, conjugate pad, nitrocellulose membrane and absorbent pad. All these parts are assembled onto a plastic card to enable the flow and get robustness. The most critical component is the nitrocellulose membrane, where the recognition elements such as antibodies, antigens or nucleotides are immobilized by means of a dispenser. The fluid test sample flows by capillarity, which means that there is no need to use pumps that require energy to run.

**Figure 1:**
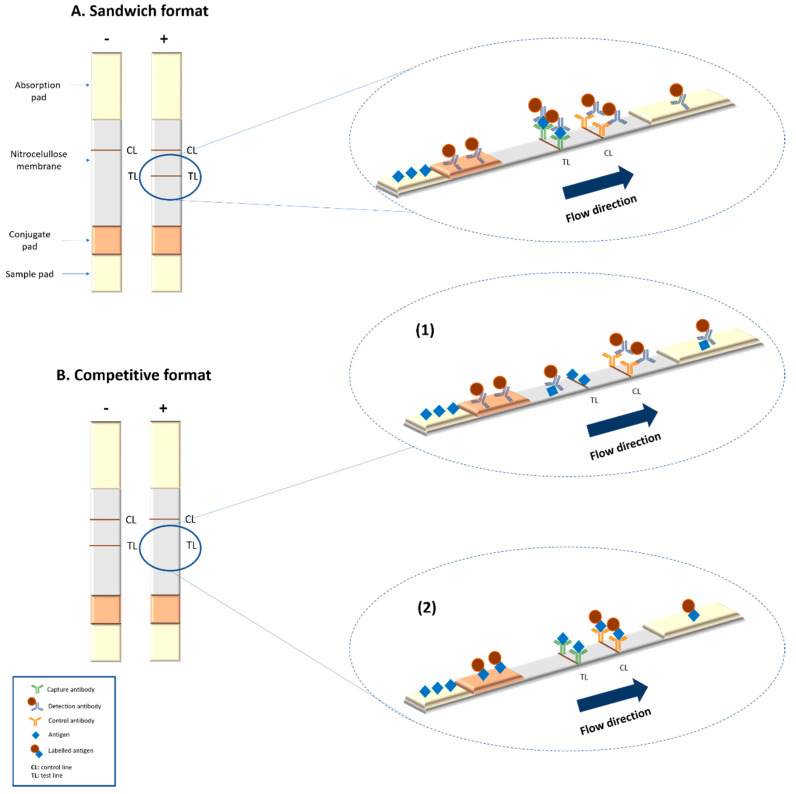
Summary of the basic formats of lateral flow assays. (**A**) Scheme of a sandwich format lateral flow immunoassays (LFIA). (**B**) Scheme of a competitive format LFIA: (1) when antigen is immobilized at the test line and labelled antibody is used as detection system; (2) when antibody is immobilized at the test line and labelled antigen is used for detection.

Different formats may be adopted in LFIA, with the sandwich format being the most common. In this assay the labelled antibody (detection antibody) forms a complex with the target analyte. These complexes will be captured at the test lines by the capture antibody immobilised on the membrane (Figure 1A). For low molecular weight molecules (e.g., haptens) the competitive format is used, where the absence of signal in the test line indicates the presence of the analyte (Figure 1B).

Nanoparticles play an important role as labels for the development of LFIA [11]. Gold nanoparticles (AuNPs) are the most popular labelling system in qualitative analysis. They display an intense red colour due to their localized surface plasmon resonance effects [12]. Latex nanoparticles are also widely employed in commercial LFIA due to their low cost [13]. They provide good performance, but the detection is not as sensitive as those achieved with other systems. Moreover, the low versatility of the latex nanoparticles prevents the development of further strategies to couple the strips to transducers.

There is a recent interest on quantitative LFIA, with the aim of achieving rapid, cheap and simple tests for biomedical or environmental applications. Limitations such as sensitivity and quantification could be overcome by the use of novel nanoparticle labels. Several nanomaterials have been used for optical, electrochemical and other emerging detection principles such as magnetic systems [11]. Fluorescent nanoparticles, which include quantum dots [14] and up-converting phosphor technologies reporter particles [15], are suitable tags to be detected by optical methods. Carbon nanoparticles [16] or nanotubes [17] have also been used with the same purpose. Colloidal selenium [18], magnetic [19], silver [20], Au@Ag core shell [21] and platinum nanoparticles [22] have been employed to improve the performance of the most traditional optical nanoparticles. Liposomes are promising multilabel systems through the encapsulation of dyes, thus enhance the sensitivity [23]. 

**Figure 2:**
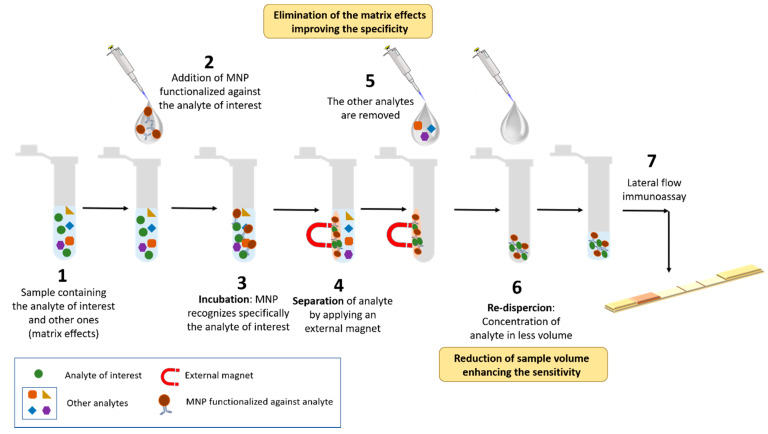
Scheme of the immunomagnetic separation (IMS) procedure.

Magnetic nanoparticles (MNP) are key players for the development of a new generation of biosensors based on LFIA. On one hand, they could be used for quantitative measurements provided that they are coupled with an external reader. On the other hand, enable IMS as well [24], which improves selectivity and sensitivity. To achieve this, MNP have to be functionalized with an active biomolecule which recognizes specifically the analyte of interest. Then, these MNP bioconjugates could be used to bind the analyte and separate it from diluted samples by applying an external magnetic field (a simple magnet could be used for many applications). The analyte-MNP complexes can be subsequently transferred to another solution and concentrated in less volume [25] as demonstrated in Figure 2.

Magnetic LFIA, or magnetic immunochromatographic assays, have been developed in different fields of interest. Despite their promising features, they have not yet been transferred to commercial in-vitro tests. In addition, review papers in this field are still scarce at the literature, probably due to the difficulties inherent to interdisciplinary research. The following sections provide a thorough analysis of the use of magnetic nanoparticles (MNP) in LFIA and their recent applications. We have firstly analysed the type of MNP and their features related to the transducers systems. Then the magnetic principles used at research and commercial devices are described, followed by a collection of the main applications of magnetic LFIA. This work aims to have an impact on the development of novel POCT biosensors based on magnetic LFIA.

## 2. Magnetic Nanoparticles as Labels in LFIA

The desired final properties of MNP vary depending on the synthesis routes employed. Synthesis protocols may be classified into three mains groups: chemical, physical and biological [26]. Chemical synthesis represents 90% of total. The main chemical routes include: coprecipitation, thermal decomposition, hydrothermal, sol-gel, polyol and microemulsion methods. Chemical synthesis can be non-aqueous or aqueous [26,27], the latter being more suitable for subsequent bioconjugation since the bioreagents have to be dispersed in water. In fact, the synthesis procedures are known to affect the outcome of the bioconjugation process, as reviewed elsewhere [28]. Once MNP are prepared, characterization of their physicochemical properties is an essential step for their control and quality [27,28]. The main parameters are shown in Table 1.

**Figure 3:**
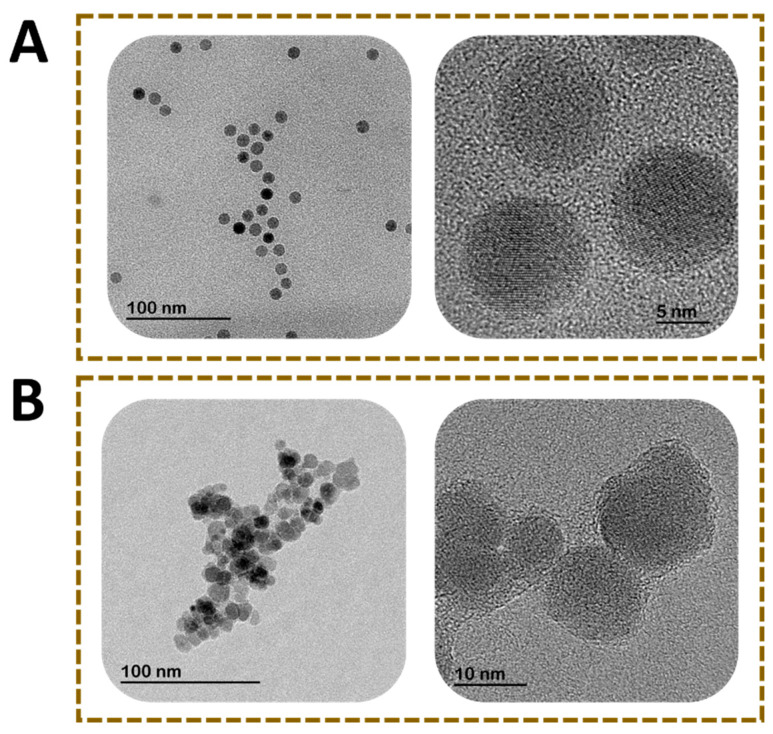
Transmission electron microscopy images of magnetite nanoparticles. (**A**) Monodisperse MNP with spherical shape and (**B**) polydisperse nanoparticles with irregular spherical shape.

In order to use MNPs as label in immunoassays, they need to be stabilized with chemical compounds holding a functional group for covalent reaction with active biomolecules (antibodies, aptamers, nucleic acids). In contrast to gold nanoparticles, the attachment of the biorecognition elements to MNPs by passive adsorption is not feasible. Various coating chemistries are therefore required for bioconjugation. The surface functional groups are a critical parameter that determines the chemical reactions and enables a proper functionalization [29]. Carboxyl-functionalized MNPs are widely used, since they easily bind the amino groups on bioreceptors and form covalent bonds by using the EDC-NHS (1-ethyl-3-(3-dimethylaminopropyl)carbodiimide/*N*-hydroxysuccinimide) chemistry. Other approach involves the coating of MNP with gold nanoparticles. These can be directly attached to biological molecules due to hydrophobic attractions, ionic interactions and dative binding [30]. The magnetic nanoparticles used for lateral flow immunoassay in the literature vary in size (10–400 nm), type of chemical coating and crystal phases. Coatings can be classified into two broad groups depending on their chemical nature: organic and inorganic [31]. These surface layers include hydroxyl, carbohydrate, thiol and phosphonate groups [26]. Magnetite and maghemite are the preferred crystal structures due to their good magnetic response, biocompatibility, facile synthesis and low-cost production [32]. The magnetic nanoparticles for LFIA are usually spherical. Figure 3 shows an example of magnetite nanoparticles. Figure 3A shows dispersed nanoparticles with a perfect spherical morphology, whereas Figure 3B shows polydisperse nanoparticle aggregates with an irregular spherical shape.

The optimal type of magnetic nanoparticles depends on the final applications and the magnetic transducing principle. The different parameter related to MNP (size, crystallinity, dispersity, magnetic properties and coating) can be controlled by the synthesis procedure. 

In recent years, the interest for the use of nanocomposites or core/shell nanoparticles has increased significantly. This kind of nanocomposites could synergistically combine the advantages of both nanoparticles [33]. Fe_3_O_4_/Au core/shell nanoparticles have been used in lateral flow immunoassay to improve the sensitivity of the assay. Gold nanoparticles can be attached to biomolecules directly, and the test line shows intense red colour. In addition, the magnetic core allows easy separation using a magnet. For this reason, Fe_3_O_4_/Au core/shell nanoparticles have been widely reported in lateral flow immunoassay as label to detect different analytes [34,35,36,37]. 

**Figure 4:**
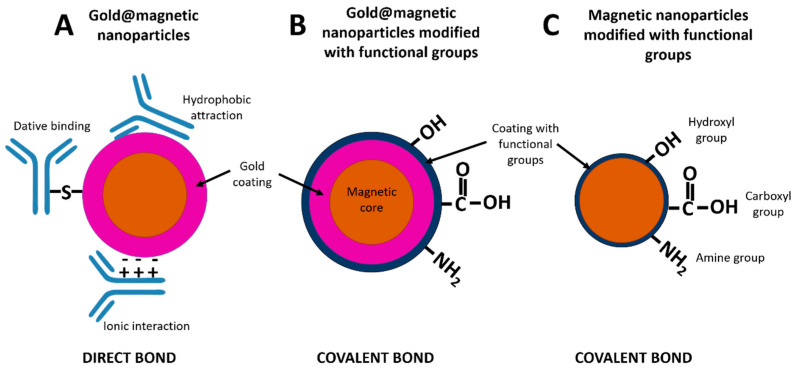
Schematic representation of direct bond between gold@magnetic nanoparticles and proteins (**A**), covalent bond between gold@magnetic nanoparticles and proteins (**B**) and covalent bond between functionalized magnetic nanoparticles and proteins (**C**).

Figure 4 shows a scheme of core/shell nanoparticles and different strategies to conjugate biorecognition elements. Gold nanoparticles are attached directly to proteins through i) hydrophobic attractions between the protein and the metal surface, ii) ionic interactions between the negatively charged nanoparticle and the positively charged sites on the protein and iii) dative binding between the metal and the conducting electrons of nitrogen and sulphur atoms of the protein (Figure 4A) [38]. In addition, gold coating could be modified with functional groups (such as hydroxyl, amine or carboxyl) to establish a covalent bond with proteins. In this case, functional groups have to be activated on the nanoparticle surface in order to attach biomolecules (Figure 4B). In contrast, MNPs have to be modified with functional groups first to enable the binding of the proteins to the particle (Figure 4C).

## 3. Detection of Magnetic Nanoparticles Used as Labels in LFIA

### 3.1. Optical Transduction

**Figure 5:**
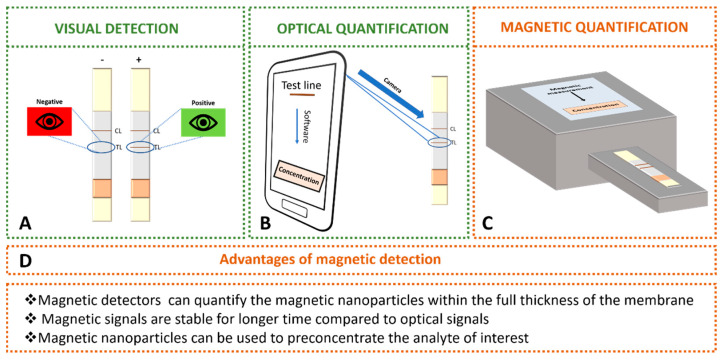
Detection of magnetic nanoparticles used as labels in LFIA: (**A**) visual detection by naked eye. (**B**) Optical detection using smartphone with special software. (**C**) Magnetic quantification using magnetic transduction. (**D**) Advantages of magnetic detection over optical detection.

Magnetic nanoparticles can be detected at the test line in LFIA by means of optical techniques (Figure 5). MNP have a molar absorption coefficient comparable with colloidal gold in the visible range [19]. For this reason, the magnetic nanoparticles are used as colorimetric labels in lateral flow immunoassays, showing a dark brown colour easily distinguishable on white nitrocellulose membranes. The optical density signal displayed by magnetic nanoparticles can be observed by naked eye (Figure 5A) or by the use of optical commercial readers and smartphones [39]. Magnetic nanoparticles aggregates have been used in order to decrease the detection limit thanks to the controlled agglomeration with poly-L-lysine [19]. The limit of detection achieved for aggregates is almost 40-fold lower than the values achieved with single dispersed magnetic nanoparticles. Yan et al. [40] have reported other advantageous strategy to enhance the optical signal (reflectance) based on the generation of magnetic network complex by means of secondary antibodies. 

Mobile phones and home-scanners have been integrated to LFIA as decentralized diagnostic tools (Figure 5B). Their simplicity and affordability make them not only a perfect candidate for devices in developing countries [41], but also an easy-to-use tool for patients requiring regular controls that may eventually reduce costs to the National Health systems. Moreover, some manufacturers provide special software, such as Novarum© or Halomic© to process strips. Applications for phone devices can provide analytical parameters for rapid tests and in simple way even in field conditions [42]. Several approaches have been reported in order to improve the limits of detection [43,44]. Ruppert et al. [43] have designed a statistical software which includes the colorimetric readout of test strip and tools for background and baseline correction. Saisin et al. [44] have optimized the camera exposure time in order to improve the sensitivity and limit of detection. They achieved enhancements up to 3-fold and 5-fold, respectively. In addition, numerous optical readers have been commercialized by several companies: Detekt Biomedical (Austin, TX, USA), Qiagen (Hilden, Germany), Axxin Inc. (Fairfield, Australia), LRE medical (Nordlingen, Germany), Abbott (Libertyville, IL, USA), Optricon (Berlin, Germany), Skannex (Oslo, Norway) and BD Company (Franklin Lakes, NJ, USA).

### 3.2. Magnetic Transduction

Magnetic detection offers several advantages over optical detection. Optical detectors only record the intensity of colour from the top layer of the membrane. However, magnetic detectors can quantify the magnetic nanoparticles within the full thickness of the membrane. In addition, the magnetic signals are stable for longer time than optical signals, and magnetic nanoparticles can be used to preconcentrate the analyte of interest.

Up to now, several methods to quantify magnetic nanoparticles at the detection zones in LFIA have been described in the literature. They are based on different physicochemical transducing principles which can be divided into two groups: magnetoresistive readers and inductive readers. Some of them are commercially available and others have been developed by academic research groups with the aim of improving the analytical characteristics or portability requirements. The different types of nanoparticles used for each transducing principle are shown in Table 1. In order to select the optimal nanoparticles, several parameters must be taken into account. The size depends on the specific applications and the transducing principle, as shown in Table 2. EDC-NHS chemistry was used for bioconjugation in all the cited works.

#### 3.2.1. Magnetoresistive LFIA Readers

Magnetoresistance (MR) is the dependence of the electrical resistivity on the applied magnetic field. Sensors based on this principle have been adapted to the dimensions of LFIA to detect the stray field fringes that the magnetic labels produce [45]. After calibration, the change in the sensor electrical resistance provides a measure of the number of particles, and consequently, the number of analytes immobilized at the strip test or control line.

**Figure 6:**
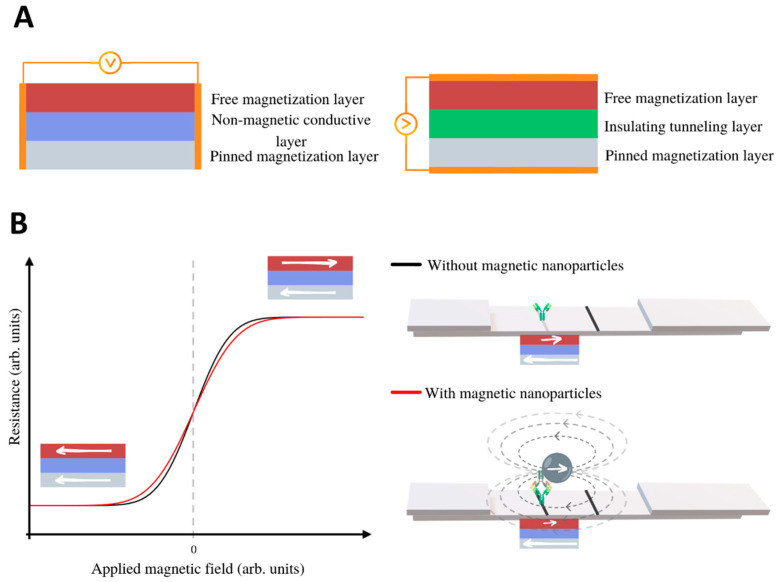
(**A**) Schemes of a Giant Magnetoresistance (GMR) sensor (left), and a Tunnel Magnetoresistance (TMR) sensor (right). (**B**) Scheme of the resistance of the MR sensor as a function of the applied field (black line). The MNP’s magnetic moments align in the direction of the applied field, and their solenoidal magnetostatic field reduces the magnetization of the free layer (the magnetic field lines are represented as grey dashed lines.) In consequence, if the applied field is positive, the resistance of the sensor decreases, and if it is negative, the resistance increases (red line).

Magnetoresistive sensors for biodetection are based on Giant MR (GMR) or Tunnel MR (TMR). GMR and TMR devices have an underlying common structure, namely, two ferromagnetic metal films separated by a non-magnetic film. The difference between the structures of these devices is in the non-magnetic spacer, which can be conductive (GMR) or insulating (TMR) (Figure 6A) [52]. The electrical resistance depends on the relative angle between the magnetization of the two ferromagnetic layers. When they are magnetized parallel, the resistance is small, and it increases for antiparallel alignment.

In GMR, the multilayers can be engineered to have parallel/antiparallel alignment in the absence of field so that the resistance is low/high [66]. Frequently, one of the layers has a pinned or fixed magnetization (meaning that it is hard to revert, either because it is a hard ferromagnet, a field-cooled antiferromagnet or because it is magnetically pinned by an additional layer.) Then, the applied field tends to modify the magnetization of the free layer, so modifying the resistance (see the black line in the graph of Figure 6B). Typically, the relative change of resistance is on the order of 5–10%. There are different types of GMR structures, depending on the non-magnetic conducting spacer and the type of magnetic layers, such as antiferromagnetic superlattices, spin valves or pseudo-spin valves.

Some research groups have developed models adapted of GMR sensors for magnetic LFIA that show an excellent potential sensitivity and spatial resolution [45,46]. The basic idea of this detection is schematized in Figure 6B: When the LFIA is placed on top of the free layer of the sensor, and the magnetic nanoreporters are magnetized by the applied field, they produce below a magnetostatic field (whose field lines are represented by dashed grey lines) that effectively decreases the magnetic field in the free layer. In consequence, the MR response in the presence of the LFIA is given by the red line in Figure 6B. Taton et al. [47] achieved detection of only 12 pg/mL of interferon gamma (IFNγ), a result that is comparable to enzyme-linked immunosorbent assays (ELISA). Serrate et al. [48] have reported a limit of detection for human chorionic gonadotropin hormone that is below the visual inspection limit (5.5 ng/mL). Ryu et al. [49] detected down to 0.01 ng/mL of the cardiac biomarker troponin I by reading out magnetic LFIA with a GMR sensor. Chicharro et al. [50] have developed a novel technique and detection architecture that include spin valves sensors, which present high sensitivity to the localized magnetic field in a single direction. It allows real-time measurements of flowing nanoparticles along the membrane, and can be used for dynamic quantification of analytes flowing through lateral flow strips [51].

In TMR (or magnetic tunnel junction MTJ) structures, the non-magnetic spacer is an electrical insulator, so thin that the electrons can tunnel through it. The tunnel crossing is more probable when both magnetizations are parallel. Then, also in this case, the applied field that produces alignment reduces the resistance. The magnetoresistance in TMR can be 5–10 times that of GMR, and the temperature drift and aging are smaller. Although they can be more sensitive, the Flicker noise coming from resistance fluctuations in TMR sensors can be larger. The literature of TMR application to LFIA reading is still scarce. Nevertheless, Lei et al. [52] have reported promising results on human chorionic gonadotropin hormone quantification.

#### 3.2.2. Inductive Readers

The magnetic sensors based on Faraday electromagnetic induction can also be used for magnetic LFIA reading. Briefly, the magnetic nanolabels are excited by an alternating magnetic field and, in turn, produce a stray magnetic field that is captured by a pick-up coil. Depending on the design, the signal is proportional to a change of self-inductance, impedance or resonance frequency of the pick-up coil. Different solutions are proposed that differ in the geometry and shape of the coils, the connection of several coils or combination of exciting frequencies [53,67].

This technology allowed Barnett et al. [54] to quantify prostate specific antigen (PSA) with a LOD of 0.8 ng/mL, and Nikitin et al. [55] to quantify small molecules such as fluorescein, biotin and chloramphenicol. Orlov et al. [56] extended the system to multiplex detection by the use of multiple pick-up coils wound around the different test lines; they simultaneously detected Botulinum Neurotoxins A, B and E in liquid samples with a LOD around 0.2 ng/mL. Among the commercial magnetic readers, the device of MagnaBioSciences (LLC, CA, USA) is most widely used in research articles, according to the number of papers [68]. This desktop device excites the particles with a C-shaped magnet, and the signal is picked up by an array of thin-film coils. It has been used, for example, for reading out LFIA for quantification of tumour markers or pathogens [57,58,59,60,61]. Gas et al. [63] have used Magnisense portable device [62] for quantification of marine toxic algae with LOD of $5\times 10^{4}$ cells/mL.

**Figure 7:**
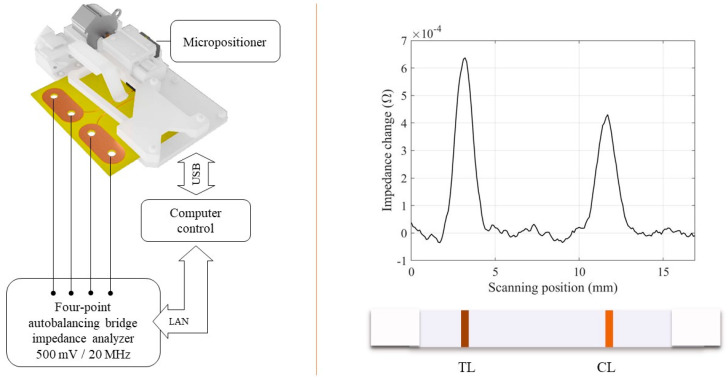
**Left**: Scheme of the scanning inductive reader for magnetic LFIA. **Right**: Signal recorded for histamine competitive LFIA (blank sample, competitive immunoassay).

Our group has developed a portable inductive sensor that scans the LFIA strip and measures the change of impedance produced by the initial magnetic permeability of the particles at radio frequency (Figure 7). In particles below the critical volume for superparamagnetic behaviour, the thermal energy has an enormous influence on the magnetization orientation. At radio frequencies, these nanoparticles are magnetically very susceptible, which can be used for their inductive detection, even without any externally applied field [69,70]. This fact reduces the complexity and size of the overall device. Mechanical positioning is done by a PLA 3D-printed micro-positioner to avoid the use of any metallic moving parts that may induce artefacts in the measurement. We have applied this technique to the quantification of PSA in the clinical range of interest [64] and histamine directly in red wine [65].

## 4. Fields of Application

### 4.1. Conventional Magnetic LFIA for Analyte Detection

Magnetic LFIAs have been used extensively in different fields: biomedicine, food, environmental control and drug monitoring. Regarding the agri-food sector, pathogen microorganisms (bacteria and virus), toxins and other hazardous molecules have been controlled and detected by magnetic LFIAs. For clinical analysis, proteins, cells and nucleic acids have been used in order to detect biomarkers, as well as hormones. In addition, the monitoring of drugs such as cocaine has been performed by means of LFIAs employing magnetic nanoparticles as labels. Table 3 and Table 4 summarize the latest magnetic LFIA reported with optical and magnetic detection, respectively.

Sandwich and competitive LFIAs are the most common formats. Table 3, Table 4 and Table 5 show several competitive LFIA for detection of molecules of interest in different fields: melamine [72], furazolidone metabolite of 3-amino-2-oxazolidinone (AOZ) [40], cocaine [39], histamine [65], thyroxine [79], unconjugated estriol (uE_3_) [82] and aflatoxin B_1_ (AFB_1_) [84]. In all cases, the competitive assays have been developed for small molecules. However, sandwich format is more suitable for larger mean size analytes such as proteins, virus or bacteria. The limits of detection for both formats are comparable, which implies that this parameter is independent of immunoassay structure. Hwang et al. [30] have reported a magnetic LFIA based on unconventional format. The test line was formed by pressing the nitrocellulose membrane to decrease its thickness instead of immobilizing specific antibodies against bacteria, as it is done at the conventional procedure. The free gold/magnetic nanoparticles passed through the pore until they reached the pressed test line. However, *Salmonella*–gold/magnetic nanoparticles complexes remained in the solution because they were too large to flow along the membrane. The flow of free gold/magnetic nanoparticles was blocked at the test line displaying the colour on spot, which was inversely proportional to the bacteria concentration.

The limit of detection reported for both systems, optical and magnetic, are similar, thus there are not significant differences when comparing similar type of analytes. However, the possibilities for MNPs combined with preconcentration steps have not yet been fully explored. The detection ranges for bacteria obtained are from 10^3^ to 10^5^ CFU/mL. For virus, it was possible to quantify levels as low as ng/mL [71] getting optical enhancement by means of double strategy based on (i) aggregation of magnetic nanoparticles with gold nanoparticles by biotin-streptavidin interactions, and (ii) pre-concentration with a magnetic field [25]. The detectable concentration for other analytes is also in the nanogram per mL range for both optical and magnetic detection, although in some case concentrations lower than nanograms have been reached for magnetic-LFIA [36,80].

It is worth remarking that gold-magnetic nanoparticles have been reported widely as alternative nanocomposite. Their use does not affect negatively to the limit of detection and they combine optical and magnetic transduction with an easy conjugation to biomolecules. In addition, they can be used for IMS.

Regarding the crystal structure of magnetic nanoparticles, magnetite (Fe_3_O_4_) has been the preferred option in order to develop LFIA. Li et al. [83] have used MnFe_2_O_4_ instead of magnetite in order to achieve stronger magnetization. They hypothesized that the hybridization of iron and manganese atoms increased the spin magnetic moment between them, resulting in a higher total magnetic moment for the nanoparticle. Carboxyl groups on the MNP surface are the preferred functional groups for the development of LFIA, as shown in Table 3, Table 4 and Table 5.

### 4.2. Immunomagnetic Separation in Combination with Other Transduction Systems

Table 3 and Table 4 show that magnetic nanoparticles have mainly been used as reporters, but they can also be used for IMS, which would lower the limit of detection in comparison with a standard LFIA. A strategy to preconcentrate the analyte from matrix or/and locate it in the detection zone (test line for LFIA) with an external magnetic field has been reported, showing the capacity to decrease the lowest detectable concentration [78]. In some applications, magnetic nanoparticles have been used to perform a double function simultaneously: separation and label for optical detection [25,35,78]. In other cases, gold and fluorescent nanoparticles have been employed in combination with magnetic nanoparticles in order to use optical nanoparticles for the transduction and magnetic nanoparticles for the IMS [85,88,89]. Nanocomposites combining magnetic nanoparticles with gold or fluorescent nanomaterials have also been reported to get the same double function [84,87]. This approach has enabled the detection of 10^0^ CFU/mL for bacteria [24] and biomarkers in picogram range [36,84].

MNP have been used for IMS in combination with other detection systems to improve sensitivity (visual, electrochemical and fluorescent), as shown in Table 5. Poonlapdecha et al. [24] preconcentrated *Campylobacter jejuni* present in poultry samples by IMS for subsequent DNA extraction and nucleic acid detection by LFIA. Li et al. [85] employed a similar procedure for detection of *Listeria monocytogenes* in lettuce samples. In addition, IMS as previous pretreatment to fluorescent LFI was employed to detect *Listeria monocytogenes* [89]. Nanocomposites containing magnetic and fluorescent nanoparticles were developed to combine IMS with fluorescence detection of toxins [84]. IMS combined with colloidal gold-based LFIA for detection of *Enterobacter cloacae* enhanced the sensitivity in comparison with conventional LFIA [88]. Other strategy that combine gold nanoparticles based LFIA with magnetic gold nanostructure for bacteria detection was developed [87]. In this case, magnetic nanostructures were used to locate the analyte at the detection zone by means of external magnet, in order to increase the reaction time for the immunoassay. The authors have also employed enzymes conjugated to gold nanoparticles to enable an extra-colour change and enhance the sensitivity.

Electrochemical transductions have been reported coupled to magnetic LFIA by means of personal blood glucose meters, which are widely used as point of care devices for people with diabetes. *Escherichia coli* was quantified by using these personal glucometers [86]. The strategy is based on the use of magnetic nanoparticles attached to invertase and antibodies against *E. coli* in order to carry out both electrochemical transduction and IMS. A different approach using these devices to quantify a biomarker of gamma-radiation exposure has recently been reported [90]. In this case, magnetic nanoparticles functionalized with specific antibodies have been used to locate the analyte in the test line with the help of a magnet. Then glucose-encapsulating liposomes were used as labels for electrochemical detection. The limit of detection obtained by personal glucose meters (Table 5) are comparable with other detection methods described previously.

Table 6 shows the limit of detection improvements by combining the detection principle with IMS.

## 5. Conclusions

The development of LFIAs using magnetic nanoparticles has emerged as a research field of great interest in the past five years. Magnetic-LFIA would enable POCT instruments to detect biomarkers, pathogens, toxins, allergens and drugs. At present, there are different magnetic transducers under research that could be coupled to LFIA. Some commercial readers, based on magnetic and optical transduction, are also available.

The control on the properties of the nanoparticles used is crucial for the development of the applications. Gold-magnetic core-shell nanoparticles have been employed as alternative to conventional nanoparticles in order to combine the properties of both materials. Nanocomposites with both materials have attracted a lot of interest to combine both the superparamagnetism of Fe_3_O_4_ and the surface chemistry of Au component. This enables an easy functionalization in addition to a simple separation. Although MNP are mainly used as labels, they can be employed for IMS as well, either as a previous step to LFIA or to concentrate the analyte at the detection zone. The most common functional group use to functionalize MNP is carboxyl group, which allows an easy conjugation to biomolecules with amine groups by carbodiimide chemistry. The magnetic nanoparticles sizes reported vary from 10 nm to 400 nm and magnetite has been the most employed crystalline structure.

The analysis of the literature reported in this review indicates that magnetic LFIAs with quantitative capability are a suitable alternative to develop quantitative biosensors for a wide range of applications.

## Figures and Tables

**Table 1:** Characterization techniques and their corresponding information obtained for magnetic nanoparticles (MNPs).

Techniques	Characterization Information
Microscopy: Transmission electron microscopy and Scanning electron microscopy	Morphology, size distribution, crystallinity and composition
X-ray diffraction (XRD)	Crystal structure and size
Dynamic light scattering (DLS)	Hydrodynamic size
Infrared spectroscopy (IR)	Nature of surface and functional groups on surface
Zeta potential	Surface charge and stability
Thermal analysis	Concentration and thermal stability
Mass spectroscopy	Concentration
Superconducting quantum interference device (SQUID)/Vibrating sample magnetometry (VSM)	Magnetic properties

**Table 2:** Type of magnetic nanoparticles used for each magnetic transducing principle.

Transducing Principle	Nanoparticles	References
**Magnetoresistive LFIA readers**	Superparamagnetic nanoparticles	[45]
	Superparamagnetic maghemite nanoparticles	[46]
	Beads of 440 nm in diameter	[47]
	Superparamagnetic Maghemite nanoparticles (200 nm)	[48]
	Magnetic beads of 200 nm	[49]
	Superparamagnetic nanoparticles (10.5 nm)	[50]
		[51]
	Superparamagnetic nanoparticles (80 nm)	[52]
**Inductive LFIA readers**	Superparamagnetic nanoparticles encapsulated in microspheres (198 nm)	[53]
	Paramagnetic particles (760 nm)	[54]
	Superparamagnetic nanoparticles (50 nm)	[55]
	Superparamagnetic nanoparticles encapsulated in microspheres (198 nm)	[56]
	Magnetic nanobeads (15, 80 and 200 nm)	[57]
	Superparamagnetic nanoparticles (140 nm)	[58]
	Superparamagnetic nanoparticles (15 nm)	[59]
	Superparamagnetic nanoparticles (140 nm)	[60]
	Colorimetric-Fluorescent-Magnetic nanospheres (300 nm)	[61]
	Superparamagnetic nanoparticles (10 nm)	[62]
	Superparamagnetic nanoparticles (200 nm)	[63]
	Superparamagnetic nanoparticles (10.5 nm)	[64]
	Superparamagnetic magnetite nanoparticles	[65]

**Table 3:** Most recent Magnetic LFIA with optical detection.

Nanoparticles	Conjugation	Detection	Analyte	Limit of Detection	Reference
**Pathogens**					
Gold magnetic nanoparticles	Via Au–S bonds	Visual detection	*Salmonella choleraesuis*	5 × 10^5^ CFU/mL	[37]
Gold magnetic nanoparticles	Via Au–S bonds	Colour intensity. ImageJ density analysis	*Salmonella*	10^3^ CFU/mL	[30]
Magnetic nanoparticles	EDC/NHS Chemistry	Colour intensity. TotalLab TL120	Potato virus X	0.5 ng/mL	[71]
Magnetic nanoparticles	EDC/NHS Chemistry	Colour intensity. TotalLab TL120	Potato virus X	0.25 ng/mL	[25]
Gold magnetic nanoparticles	Via Au–S bonds	Visual detection	Avian influenza virus subtype H7 (AIV H7)	10^3^ ^5^ EID_50_	[33]
**Toxins and allergens**					
Gold magnetic nanoparticles	Via Au–S bonds	Visual detection	β-Conglutin	8 fM	[35]
Magnetic nanoparticles	EDC/NHS Chemistry	Visual detection	Melamine	0.4 ppm for Fe_2_O_3_PEG 2.2 ppm for Fe_3_O_4_-PEG	[72]
Magnetic nanoparticles	EDC/NHS Chemistry	Visual detection	Furazolidone metabolite of 3-amino-2-oxazolidinone (AOZ)	0.044 ng/mL	[40]
**Biomarkers**					
Magnetic nanoparticles	EDC chemistry	Visual detection	Carcinoembryonic antigen (CEA)	0.25 ng/mL	[73]
Magnetic nanoparticles	EDC/NHS Chemistry	Reflectance measurements. ESE Quant LR3 (Qiagen Inc., Germany)	Extracellular vesicles (EVs)	10^7^ EVs/mL	[74]
Gold magnetic nanoparticles	EDC chemistry	Visual detection	Genotyping of MTHFR C677T	5 ng	[75]
Gold magnetic nanoparticles	CTAB-PSS modification (direct bonds)	Visual detection	Genotype Apolipoprotein E	10 ng	[76]
Gold magnetic nanoparticles	EDC chemistry	Visual detection	IgM class antibodies related infections	-	[77]
Magnetic nanoparticles	Periodate-based oxidation of the glycosylated Fc residues	Colour intensity. ImageJ density analysis	Human chorionic gonadotropin (hCG)	0.31 ng/mL	[78]
**Drugs**					
Magnetic nanoparticles	EDC/NHS Chemistry	Smart phone camera was used for quantitative analysis	Cocaine	5 ng/mL	[39]

**Table 4:** Most recent Magnetic LFIA with magnetic detection.

Nanoparticles	Conjugation	Detection	Analyte	Limit of Detection	Reference
**Pathogens**					
Magnetic nanoparticles	EDC/NHS Chemistry	MAR system (MagnaBioSciences, CA, USA)	*Vibrio parahaemolyticus*	4.73 × 10^3^ CFU/mL	[60]
Colorimetric-Fluorescent-Magnetic nanoparticles	EDC/NHS Chemistry	MAR system (MagnaBioSciences, CA, USA) Fibre optic spectrometer	*Salmonella typhimurium*	1.88 × 10^4^ CFU/mL: naked detection 3.75 × 10^3^ CFU/mL: magnetic and fluorescent detection	[61]
Magnetic nanoparticles	EDC/NHS Chemistry	Reader Miateks (Magnisense)	*Alexandrium minutum*	10^5^ cells/L	[63]
**Toxins and allergens**					
Magnetic nanoparticles	EDC/NHS Chemistry	Magnetic particle quantification (MPQ) method	Botulinum neurotoxin (BoNT) types A, B and E	0.22 ng/mL for BoNT-A 0.11 ng/mL for BoNT-B 0.32 ng/mL for BoNT-E	[56]
Magnetic nanoparticles	EDC/NHS Chemistry	Novel sensor developed by authors Reflectance measurements. ESE Quant LR3 (Qiagen Inc., Germany)	Histamine	1.2 mg/L for magnetic sensor 1.5 mg/L for optical reader	[65]
**Biomarkers**					
Magnetic nanoparticles	EDC/NHS Chemistry	MAR system (MagnaBioSciences, CA, USA)	Carbohydrate antigen 72-4 (CA72-4)	0.38 IU/mL	[59]
Magnetic nanoparticles	EDC/NHS Chemistry	Novel sensor developed by authors Reflectance measurements. ESE Quant LR3 (Qiagen Inc., Germany)	Prostate-Specific Antigen	0.25 ng/mL	[64]
Magnetic nanoparticles	EDC/NHS Chemistry	MAR system (MagnaBioSciences, CA, USA)	Neuron specific enolase (NSE) carcinoembryonic antigen (CEA).	0.094 ng/mL for NSE 0.045 ng/mL for CEA	[57]
Magnetic nanoparticles	EDC/NHS Chemistry	Magnetic particle quantification (MPQ) method	Thyroxine	20 fM	[79]
Magnetic nanoparticles	EDC Chemistry	MAR system (MagnaBioSciences, CA, USA)	Amino-terminal pro-B-type natriuretic peptide (NT-proBNP)	100 pg/mL	[80]
Gold magnetic nanoparticles	EDC Chemistry	MAR system (MagnaBioSciences, CA, USA)	Single nucleotide polymorphisms (SNPs)	0.04 pg/μL with plasmid	[36]
Magnetic nanoparticles	EDC/NHS Chemistry	MIR system developed by authors	Troponin I (cTnI) Creatine kinase isoenzyme MB (CKMB) Myoglobin (Myo)	0.0089 ng/mL for cTnI 0.063 ng/mL for CKMB 0.05 ng/mL for Myo	[81]
Magnetic nanoparticles	EDC/NHS Chemistry	MAR system (MagnaBioSciences, CA, USA)	Unconjugated estriol (uE3)	0.86 nmol/L	[82]
Magnetic nanoparticles	EDC/NHS Chemistry	MAR system (MagnaBioSciences, CA, USA)	D-dimer	0.05 μg/mL	[83]
Gold magnetic nanoparticles	-	Magnetic quantitative immunoanalyzer	C-reactive protein (CRP)	0.15 mg/mL	[34]

**Table 5:** Other detection and magnetic nanoparticles used for immunomagnetic separation.

Nanoparticles	Conjugation	Detection	Analyte	Limit of Detection	Reference
Magnetic nanoparticles	Glutaraldehyde chemistry	Visual detection	*Campylobacter jejuni*	10^0^ with pure culture 10^1^ with poultry samples	[24]
Magnetic nanoparticles	Biotin-streptavidin affinity	Visual detection	*Listeria monocytogenes*	3.5 × 10^3^ CFU/mL for standards 3.5 × 10^4^ CFU/g in real samples	[85]
Magnetic nanoparticles	EDC/NHS Chemistry	Electrochemical detection. Glucose meter	*Escherichia coli O157:H7*	6.2 × 10^4^ CFU/mL	[86]
Gold magnetic nanoparticles	Via Au–S bonds	Visual detection	*Escherichia coli O157:H7* and *Salmonella typhimurium*	23 CFU/mL for E. coli 17 CFU/mL for Salmonella	[87]
Magnetic nanoparticles	Glutaraldehyde chemistry	Visual detection.	*Enterobacter cloacae*	10^2^ CFU/mL	[88]
Magnetic nanoparticles	EDC/NHS Chemistry	Fluorescent detection	*Listeria monocytogenes*	10^4^ CFU/mL	[89]
Fluorescent magnetic nanoparticles	EDC chemistry	Fluorescent detection. Fluorescent strip reader (Suzhou Hemai Precision Instrument Co., Ltd. Jiangsu, China).	Aflatoxin B_1_ (AFB_1_)	3 pg/mL in sauce extract 51 pg/mL in real dark soy sauce	[84]
Magnetic nanoparticles	EDC/NHS Chemistry	Electrochemical detection. Glucose meter	Phospho-p53^15^	50 pg/mL	[90]

**Table 6:** Limit of detection improvements using IMS.

Analyte	Limit of Detection Using LFIA	Limit of Detection Using LFIA in Combination with IMS	References
**Gold nanoparticles as label**			
*Campylobacter jejuni*	10^5^ cfu/mL for gold nanoparticles (pure culture) 10^4^ cfu/mL for quantum dots (pure culture)	10^0^ cfu/mL (pure culture) 10^1^ cfu/mL (poultry sample)	[24,91,92]
*Listeria monocytogenes*	10^4^ cfu/mL for superparamagnetic nanoparticles 3.7 × 10^6^ cfu/mL for gold nanoparticles	3.5 × 10^3^ cfu/mL (buffer) 3.5 × 10^4^ cfu/g (lettuce samples)	[58,85,93]
*E. coli O157:H7* and *Salmonella typhimurium*	*E. coli O157:H7* 10^5^ cfu/mL for gold nanoparticles 10^4^ cfu/mL for fluorescent microspheres *Salmonella typhimurium* 10^4^ cfu/mL for gold nanoparticles	23 CFU/mL for *E. coli* 17 CFU/mL for *Salmonella*	[87,94,95]
*Potato virus X*	0.25 ng/mL for combination of magnetic nanoparticles with gold nanoparticles	8 ng/mL	[25]
*Enterobacter cloacae*	10^3^ cfu/mL for gold nanoparticles	10^2^ cfu/mL	[88]
β-conglutin	5 nM for gold nanoparticles	8 fM	[35]
**Fluorescent nanoparticles**			
Aflatoxin B1	10 µg/mL for gold nanoparticles 0.1 ng/mL for silver@gold nanoparticles	3 pg/mL in sauce extract 51 pg/mL in real dark soy sauce	[84,96,97]
*Listeria monocytogenes*	3.7 × 10^6^ cfu/mL for gold nanoparticles	10^4^ CFU/mL	[89,93]
**Electrochemical detection**			
*Escherichia coli O157:H7*	10^5^ cfu/mL for gold nanoparticles 10^4^ cfu/mL for fluorescent microspheres	6.2 × 10^4^ CFU/mL	[86,95]

## References

[1] Vashist S.K., Luppa P.B., Yeo L.Y., Ozcan A., Luong J.H.T. (2015). Emerging technologies for next-generation point-of-care testing. Trends Biotechnol..

[2] Klonoff D.C. (2014). Point-of-care blood glucose meter accuracy in the hospital setting. Diabetes Spectr..

[3] Dincer C., Bruch R., Kling A., Dittrich P.S., Urban G.A. (2017). Multiplexed point-of-care testing—xPOCT. Trends Biotechnol..

[4] Yager P., Domingo G.J., Gerdes J. (2008). Point-of-care diagnostics for global health. Annu. Rev. Biomed. Eng..

[5] Choi J.R., Yong K.W., Choi J.Y., Cowie A.C. (2019). Emerging point-of-care technologies for food safety analysis. Sensors.

[6] Lau H.Y., Botella J.R. (2017). Advanced DNA-based point-of-care diagnostic methods for plant diseases detection. Front. Plant Sci..

[7] Cummins B.M., Ligler F.S., Walker G.M. (2016). Point-of-care diagnostics for niche applications. Biotechnol. Adv..

[8] Luzzi V. (2019). Point of Care Devices for Drugs of Abuse Testing.

[9] Mandal N., Mitra S., Bandyopadhyay D. (2019). Paper-sensors for point-of-care monitoring of drinking water quality. IEEE Sens. J..

[10] Li Z., Yi Y., Luo X., Xiong N., Liu Y., Li S., Sun R., Wang Y., Hu B., Chen W. (2020). Development and clinical application of a rapid IgM-IgG combined antibody test for SARS-CoV-2 infection diagnosis. J. Med. Virol..

[11] Quesada-González D., Merkoçi A. (2015). Nanoparticle-based lateral flow biosensors. Biosens. Bioelectron..

[12] Petrakova A.V., Urusov A.E., Zherdev A.V., Dzantiev B.B. (2019). Gold nanoparticles of different shape for bicolor lateral flow test. Anal. Biochem..

[13] https://www.expedeon.com/resources/applications/lateral-flow-immunoassay/.

[14] Sotnikov D.V., Barshevskaya L.V., Zherdev A.V. (2020). Immunochromatographic system for serodiagnostics of cattle brucellosis using gold nanoparticles and signal amplification with quantum dots. Appl. Sci..

[15] Yang X., Liu L., Hao Q., Zou D., Zhang X., Zhang L., Li H., Qiao Y., Zhao H., Zhou L. (2017). Development and evaluation of Up-Converting phosphor technology-based lateral flow assay for quantitative detection of NT-proBNP in blood. PLoS ONE.

[16] Aktas G.B., Wichers J.H., Skouridou V., van Amerongen A., Masip L. (2019). Nucleic acid lateral flow assays using a conjugate of a DNA binding protein and carbon nanoparticles. Microchim. Acta.

[17] Qiu W., Baryeh K., Takalkar S., Chen W., Liu G. (2019). Carbon nanotube-based lateral flow immunoassay for ultrasensitive detection of proteins: Application to the determination of IgG. Microchim. Acta.

[18] Wang Z., Jing J., Ren Y., Guo Y., Tao N., Zhou Q., Zhang H., Ma Y., Wang Y. (2019). Preparation and application of selenium nanoparticles in a lateral flow immunoassay for clenbuterol detection. Mater. Lett..

[19] Liu C., Jia Q., Yang C., Qiao R., Jing L., Wang L., Xu C., Gao M. (2011). Lateral flow immunochromatographic assay for sensitive pesticide detection by using Fe_3_O_4_ nanoparticle aggregates as color reagents. Anal. Chem..

[20] Rodríguez M.O., Covián L.B., García A.C., Blanco-López M.C. (2016). Silver and gold enhancement methods for lateral flow immunoassays. Talanta.

[21] Blanco-Covián L., Montes-García V., Girard A., Fernández-Abedul M.T., Pérez-Juste J., Pastoriza-Santos I., Faulds K., Graham D., Blanco-López M.C. (2017). Au@Ag SERRS tags coupled to a lateral flow immunoassay for the sensitive detection of pneumolysin. Nanoscale.

[22] Park J.M., Jung H.W., Chang Y.W., Kim H.S., Kang M.J., Pyun J.C. (2015). Chemiluminescence lateral flow immunoassay based on Pt nanoparticle with peroxidase activity. Anal. Chim. Acta.

[23] Leem H., Shukla S., Song X., Heu S., Kim M. (2014). An Efficient Liposome-Based Immunochromatographic Strip Assay for the Sensitive Detection of SalmonellaTyphimurium in Pure Culture. J. Food Saf..

[24] Poonlapdecha W., Seetang-Nun Y., Wonglumsom W., Tuitemwong K., Erickson L.E., Hansen R.R., Tuitemwong P. (2018). Antibody-conjugated ferromagnetic nanoparticles with lateral flow test strip assay for rapid detection of Campylobacter jejuni in poultry samples. Int. J. Food Microbiol..

[25] Razo S.C., Panferov V.G., Safenkova I.V., Varitsev Y.A., Zherdev A.V., Dzantiev B.B. (2018). Double-enhanced lateral flow immunoassay for potato virus X based on a combination of magnetic and gold nanoparticles. Anal. Chim. Acta.

[26] Anwar S., Khawja M., Ficiar E., Ruffinatti F.A., Stura I., Argenziano M., Abollino O., Cavalli R., Guiot C., Agata F.D. (2019). Magnetic iron oxide nanoparticles: Synthesis, characterization and functionalization for biomedical applications in the central nervous system. Materials.

[27] Mourdikoudis S., Pallares R.M., Thanh N.T.K. (2018). Characterization techniques for nanoparticles: Comparison and complementarity upon studying nanoparticle properties. Nanoscale.

[28] Sandler S.E., Fellows B., Thompson Mefford O. (2019). Best practices for characterization of magnetic nanoparticles for biomedical applications. Anal. Chem..

[29] Du J., Zhao Y., Yang Z., Xu C., Lu Y., Pan Y., Shi D., Wang Y. (2016). Influence of controlled surface functionalization of magnetic nanocomposites on the detection performance of immunochromatographic test. Sens. Actuators B Chem..

[30] Hwang J., Kwon D., Lee S., Jeon S. (2016). Detection of: Salmonella bacteria in milk using gold-coated magnetic nanoparticle clusters and lateral flow filters. RSC Adv..

[31] Chen Z., Wu C., Zhang Z., Wu W., Wang X., Yu Z. (2018). Synthesis, functionalization, and nanomedical applications of functional magnetic nanoparticles. Chin. Chem. Lett..

[32] Wu W., Wu Z., Yu T., Jiang C., Kim W.S. (2015). Recent progress on magnetic iron oxide nanoparticles: Synthesis, surface functional strategies and biomedical applications. Sci. Technol. Adv. Mater..

[33] Huang J., Xie Z., Xie L., Xie Z., Luo S., Deng X., Huang L., Zeng T., Zhang Y., Wang S. (2018). Au/ Fe_3_O_4_ core-shell nanoparticles are an efficient immunochromatography test strip performance enhancer—A comparative study with Au and Fe3O4 nanoparticles. RSC Adv..

[34] Zhang L., Zhang Q., Gao M., Luo Z., Zhang Y., Li X., Hua K., Zhang C., Lai W., Cui Y. (2019). Clinical experimental study of GoldMag^®^ immunochromatography in high sensitive C reactive protein detection from whole blood and plasma. J. Magn. Magn. Mater..

[35] Wu Z., He D., Xu E., Jiao A., Chughtai M.F.J., Jin Z. (2018). Rapid detection of β-conglutin with a novel lateral flow aptasensor assisted by immunomagnetic enrichment and enzyme signal amplification. Food Chem..

[36] Liu X., Zhang C., Liu K., Wang H., Lu C., Li H., Hua K., Zhu J., Hui W., Cui Y. (2018). Multiple SNPs detection based on lateral flow assay for phenylketonuria diagnostic. Anal. Chem..

[37] Xia S., Yu Z., Liu D., Xu C., Lai W. (2016). Developing a novel immunochromatographic test strip with gold magnetic bifunctional nanobeads (GMBN) for efficient detection of Salmonella choleraesuis in milk. Food Control.

[38] Thobhani S., Attree S., Boyd R., Kumarswami N., Noble J., Szymanski M., Porter R.A. (2010). Bioconjugation and characterisation of gold colloid-labelled proteins. J. Immunol. Methods.

[39] Wu J., Dong M., Zhang C., Wang Y., Xie M., Chen Y. (2017). Magnetic lateral flow strip for the detection of cocaine in urine by naked eyes and smart phone camera. Sensors.

[40] Yan L., Dou L., Bu T., Huang Q., Wang R., Yang Q., Huang L., Wang J., Zhang D. (2018). Highly sensitive furazolidone monitoring in milk by a signal amplified lateral flow assay based on magnetite nanoparticles labeled dual-probe. Food Chem..

[41] Pilavaki E., Demosthenous A. (2017). Optimized lateral flow immunoassay reader for the detection of infectious diseases in developing countries. Sensors.

[42] Eltzov E., Guttel S., Low Yuen Kei A., Sinawang P.D., Ionescu R.E., Marks R.S. (2015). Lateral flow immunoassays - from paper strip to smartphone technology. Electroanalysis.

[43] Ruppert C., Phogat N., Laufer S., Kohl M., Deigner H.P. (2019). A smartphone readout system for gold nanoparticle-based lateral flow assays: Application to monitoring of digoxigenin. Microchim. Acta.

[44] Saisin L., Amarit R., Somboonkaew A., Gajanandana O., Himananto O., Sutapun B. (2018). Significant sensitivity improvement for camera-based lateral flow immunoassay readers. Sensors.

[45] Park J. (2016). A giant magnetoresistive reader platform for quantitative lateral flow immunoassays. Sens. Actuators A Phys..

[46] Marquina C., De Teresa J.M., Serrate D., Marzo J., Cardoso F.A., Saurel D., Cardoso S., Freitas P.P., Ibarra M.R. (2012). GMR sensors and magnetic nanoparticles for immuno-chromatographic assays. J. Magn. Magn. Mater..

[47] Taton K., Johnson D., Guire P., Lange E., Tondra M. (2009). Lateral flow immunoassay using magnetoresistive sensors. J. Magn. Magn. Mater..

[48] Serrate D., De Teresa J.M., Marquina C., Marzo J., Saurel D., Cardoso F.A., Cardoso S., Freitas P.P., Ibarra M.R. (2012). Quantitative biomolecular sensing station based on magnetoresistive patterned arrays. Biosens. Bioelectron..

[49] Ryu Y., Jin Z., Kang M.S., Kim H.S. (2011). Increase in the detection sensitivity of a lateral flow assay for a cardiac marker by oriented immobilization of antibody. Biochip J..

[50] Chicharo A., Cardoso F., Cardoso S., Freitas P.P. (2014). Dynamical detection of magnetic nanoparticles in paper microfluidics with spin valve sensors for point-of-care applications. IEEE Trans. Magn..

[51] Chicharo A., Cardoso F., Cardoso S., Freitas P.J.P. (2015). Real-time monitoring of magnetic nanoparticles diffusion in lateral flow microporous membrane using spin valve sensors. IEEE Trans. Magn..

[52] Lei H., Wang K., Ji X., Cui D. (2016). Contactless measurement of magnetic nanoparticles on lateral flow strips using tunneling magnetoresistance (TMR) sensors in differential configuration. Sensors.

[53] Guteneva N.V., Znoyko S.L., Orlov A.V., Nikitin M.P., Nikitin P.I. (2018). Volumetric registration of magnetic nanoparticles for optimization of quantitative immunochromatographic assays for detection of small molecules. EPJ Web Conf..

[54] Barnett J.M., Wraith P., Kiely J., Persad R., Hurley K., Hawkins P., Luxton R. (2014). An inexpensive, fast and sensitive quantitative lateral flow magneto-immunoassay for total prostate specific antigen. Biosensors.

[55] Nikitin M.P., Orlov A.V., Znoyko S.L., Bragina V.A., Gorshkov B.G., Ksenevich T.I., Cherkasov V.R., Nikitin P.I. (2018). Multiplex biosensing with highly sensitive magnetic nanoparticle quantification method. J. Magn. Magn. Mater..

[56] Orlov A.V., Znoyko S.L., Cherkasov V.R., Nikitin M.P., Nikitin P.I. (2016). Multiplex Biosensing Based on Highly Sensitive Magnetic Nanolabel Quantification: Rapid Detection of Botulinum Neurotoxins A, B, and e in Liquids. Anal. Chem..

[57] Lu W., Wang K., Xiao K., Qin W., Hou Y., Xu H., Yan X., Chen Y., Cui D., He J. (2017). Dual Immunomagnetic Nanobeads-Based Lateral Flow Test Strip for Simultaneous Quantitative Detection of Carcinoembryonic Antigen and Neuron Specific Enolase. Sci. Rep..

[58] Shi L., Wu F., Wen Y., Zhao F., Xiang J., Ma L. (2015). A novel method to detect Listeria monocytogenes via superparamagnetic lateral flow immunoassay. Anal. Bioanal. Chem..

[59] Chen Y., Wang K., Liu Z., Sun R., Cui D., He J. (2016). Rapid detection and quantification of tumor marker carbohydrate antigen 72-4 (CA72-4) using a superparamagnetic immunochromatographic strip. Anal. Bioanal. Chem..

[60] Liu Y., Zhang Z., Wang Y., Zhao Y., Lu Y., Xu X., Yan J., Pan Y. (2015). A highly sensitive and flexible magnetic nanoprobe labeled immunochromatographic assay platform for pathogen Vibrio parahaemolyticus. Int. J. Food Microbiol..

[61] Hu J., Jiang Y.Z., Tang M., Wu L.L., Xie H.Y., Zhang Z.L., Pang D.W. (2019). Colorimetric-fluorescent-magnetic nanosphere-based multimodal assay platform for salmonella detection. Anal. Chem..

[62] Motte L., Benyettou F., De Beaucorps C., Lecouvey M., Milesovic I., Lalatonne Y. (2011). Multimodal superparamagnetic nanoplatform for clinical applications: Immunoassays, imaging & therapy. Faraday Discuss..

[63] Gas F., Baus B., Queré J., Chapelle A., Dreanno C. (2016). Rapid detection and quantification of the marine toxic algae, Alexandrium minutum, using a super-paramagnetic immunochromatographic strip test. Talanta.

[64] Lago-Cachón D., Oliveira-Rodríguez M., Rivas M., Blanco-López M.C., Martínez-García J.C., Moyano A., Salvador M., García J.A. (2017). Scanning Magneto-Inductive Sensor for Quantitative Assay of Prostate-Specific Antigen. IEEE Magn. Lett..

[65] Moyano A., Salvador M., Martínez-García J.C., Socoliuc V., Vékás L., Peddis D., Alvarez M.A., Fernández M., Rivas M., Blanco-López M.C. (2019). Magnetic immunochromatographic test for histamine detection in wine. Anal. Bioanal. Chem..

[66] Hall D.A., Gaster R.S., Lin T., Osterfeld S.J., Han S., Murmann B., Wang S.X. (2010). GMR biosensor arrays: A system perspective. Biosens. Bioelectron..

[67] Makiranta J.J., Lekkala J.O. Modeling and Simulation of Magnetic Nanoparticle Sensor. Proceedings of the 2005 IEEE Engineering in Medicine and Biology 27th Annual Conference.

[68] MagnaBioSciences, LLC http://www.magnabiosciences.com/.

[69] Lago-Cachón D., Rivas M., Martínez-García J.C., García J.A. (2013). Cu impedance-based detection of superparamagnetic nanoparticles. Nanotechnology.

[70] Rivas M., Lago-Cachón D., Martínez-García J.C., García J.A., Calleja A.J. (2014). Eddy-current sensing of superparamagnetic nanoparticles with spiral-like copper circuits. Sens. Actuators A Phys..

[71] Panferov V.G., Safenkova I.V., Zherdev A.V., Dzantiev B.B. (2017). Setting up the cut-off level of a sensitive barcode lateral flow assay with magnetic nanoparticles. Talanta.

[72] Huang W.-C., Wu K.-H., Hung H.-C., Wang J.-C., Chang S.-C. (2018). Magnetic nanoparticle-based lateral flow immunochromatographic strip as a reporter for rapid detection of melamine. J. Nanosci. Nanotechnol..

[73] Liu F., Zhang H., Wu Z., Dong H., Zhou L., Yang D., Ge Y., Jia C., Liu H., Jin Q. (2016). Highly sensitive and selective lateral flow immunoassay based on magnetic nanoparticles for quantitative detection of carcinoembryonic antigen. Talanta.

[74] Oliveira-Rodríguez M., Serrano-Pertierra E., García A.C., Martín S.L., Mo M.Y., Cernuda-Morollón E., Blanco-López M.C. (2017). Point-of-care detection of extracellular vesicles: Sensitivity optimization and multiple-target detection. Biosens. Bioelectron..

[75] Hui W., Zhang S., Zhang C., Wan Y., Zhu J., Zhao G., Wu S., Xi D., Zhang Q., Li N. (2016). A novel lateral flow assay based on GoldMag nanoparticles and its clinical applications for genotyping of MTHFR C677T polymorphisms. Nanoscale.

[76] Lian T., Hui W., Li X., Zhang C., Zhu J., Li R., Wan Y., Cui Y. (2016). Apolipoprotein e genotyping using PCR-GoldMag lateral flow assay and its clinical applications. Mol. Med. Rep..

[77] Li X., Zhang Q., Hou P., Chen M., Hui W., Vermorken A., Luo Z., Li H., Li Q., Cui Y. (2015). Gold magnetic nanoparticle conjugate-based lateral flow assay for the detection of IgM class antibodies related to TORCH infections. Int. J. Mol. Med..

[78] Jacinto M.J., Trabuco J.R.C., Vu B.V., Garvey G., Khodadady M., Azevedo A.M., Aires-Barros M.R., Chang L., Kourentzi K., Litvinov D. (2018). Enhancement of lateral flow assay performance by electromagnetic relocation of reporter particles. PLoS ONE.

[79] Znoyko S.L., Orlov A.V., Pushkarev A.V., Mochalova E.N., Guteneva N.V., Lunin A.V., Nikitin M.P., Nikitin P.I. (2018). Ultrasensitive quantitative detection of small molecules with rapid lateral-flow assay based on high-affinity bifunctional ligand and magnetic nanolabels. Anal. Chim. Acta.

[80] Yang D., Ma J., Xue C., Wang L., Wang X. (2018). One-pot synthesis of poly (acrylic acid)-stabilized Fe_3_O_4_ nanocrystal clusters for the simultaneously qualitative and quantitative detection of biomarkers in lateral flow immunoassay. J. Pharm. Biomed. Anal..

[81] Hong L., Wang K., Yan W., Xu H., Chen Q., Zhang Y., Cui D., Jin Q., He J. (2018). High performance immunochromatographic assay for simultaneous quantitative detection of multiplex cardiac markers based on magnetic nanobeads. Theranostics.

[82] Wang C., Guan D., Chen C., He S., Liu X., Wang C., Wu H. (2018). Rapid detection of unconjugated estriol in the serum via superparamagnetic lateral flow immunochromatographic assay. Anal. Bioanal. Chem..

[83] Li X., Wang Y., Tang Q., Li Q. (2019). MnFe 2 O 4 nanoclusters as labels for the quantitative detection of D-dimer in a lateral-flow immunochromatographic assay. J. Chinese Chem. Soc..

[84] Guo L., Shao Y., Duan H., Ma W., Leng Y., Huang X., Xiong Y. (2019). Magnetic Quantum Dot Nanobead-Based Fluorescent Immunochromatographic Assay for the Highly Sensitive Detection of Aflatoxin B 1 in Dark Soy Sauce. Anal. Chem..

[85] Li F., Li F., Luo D., Lai W., Xiong Y., Xu H. (2018). Biotin-exposure-based immunomagnetic separation coupled with nucleic acid lateral flow biosensor for visibly detecting viable Listeria monocytogenes. Anal. Chim. Acta.

[86] Huang H., Zhao G., Dou W. (2018). Portable and quantitative point-of-care monitoring of Escherichia coli O157:H7 using a personal glucose meter based on immunochromatographic assay. Biosens. Bioelectron..

[87] Ren W., Cho I.H., Zhou Z., Irudayaraj J. (2016). Ultrasensitive detection of microbial cells using magnetic focus enhanced lateral flow sensors. Chem. Commun..

[88] Zhang X., Zhou J., Zhang C., Zhang D., Su X. (2016). Rapid detection of Enterobacter cloacae by immunomagnetic separation and a colloidal gold-based immunochromatographic assay. RSC Adv..

[89] Li Q., Zhang S., Cai Y., Yang Y., Hu F., Liu X., He X. (2017). Rapid detection of Listeria monocytogenes using fluorescence immunochromatographic assay combined with immunomagnetic separation technique. Int. J. Food Sci. Technol..

[90] Zhao Y., Chen X., Lin S., Du D., Lin Y. (2017). Integrated immunochromatographic strip with glucometer readout for rapid quantification of phosphorylated proteins. Anal. Chim. Acta.

[91] Xu F., Xu D., Ming X., Xu H., Li B., Li P., Aguilar Z.P., Cheng T., Wu X., Wei H. (2013). Quantum dot-based immunochromatography test strip for rapid detection of campylobacter jejuni. J. Nanosci. Nanotechnol..

[92] Xu D., Wu X., Li B., Li P., Ming X., Chen T., Wei H., Xu F. (2013). Rapid detection of Campylobacter jejuni using fluorescent microspheres as label for immunochromatographic strip test. Food Sci. Biotechnol..

[93] Wang W., Liu L., Song S., Xu L., Kuang H., Zhu J., Xu C. (2017). Identification and quantification of eight Listeria monocytogene serotypes from Listeria spp. using a gold nanoparticle-based lateral flow assay. Microchim. Acta.

[94] Moongkarndi P., Rodpai E., Kanarat S. (2011). Evaluation of an immunochromatographic assay for rapid detection of salmonella enterica serovars typhimurium and enteritidis. J. Vet. Diagn. Investig..

[95] Xie Q.Y., Wu Y.H., Xiong Q.R., Xu H.Y., Xiong Y.H., Liu K., Jin Y., Lai W.H. (2014). Advantages of fluorescent microspheres compared with colloidal gold as a label in immunochromatographic lateral flow assays. Biosens. Bioelectron..

[96] Liao J.Y., Li H. (2010). Lateral flow immunodipstick for visual detection of aflatoxin B1 in food using immuno-nanoparticles composed of a silver core and a gold shell. Microchim. Acta.

[97] Moon J., Kim G., Lee S. (2012). A Gold nanoparticle and aflatoxin B1-BSA conjugates based lateral flow assay method for the analysis of aflatoxin B1. Materials.

